# A228 PSYCHOSOCIAL FACTORS IN ADULTHOOD AND RISK OF INFLAMMATORY BOWEL DISEASE

**DOI:** 10.1093/jcag/gwae059.228

**Published:** 2025-02-10

**Authors:** C Lanoix, C Fantodji, P Jantchou, M Rousseau

**Affiliations:** Universite de Montreal, Montreal, QC, Canada; Institut national de la recherche scientifique, Quebec, QC, Canada; Centre Hospitalier Universitaire Sainte-Justine, Montreal, QC, Canada; Institut national de la recherche scientifique, Quebec, QC, Canada

## Abstract

**Background:**

Few studies have assessed the possible etiological role of psychosocial factors on inflammatory bowel disease (IBD). We have previously reported that some childhood psychosocial factors were associated with increased odds of IBD in adulthood.

**Aims:**

The aim of this study was to estimate the associations between self-reported psychosocial factors first experienced in adulthood and occurrence of IBD later in life.

**Methods:**

This matched case-control study was nested within a cohort of individuals born in Quebec in 1970-1974. Cases were identified with validated algorithms based on health services from 1983 to 2014; their date of diagnosis was based on their first health service (index date). Only cases diagnosed with Crohn’s disease (CD) (n = 1041) or ulcerative colitis (UC) (n = 511) at ≥18 years were included. Each case was individually matched with a control based on sex and birth year. Cases and controls completed a questionnaire documenting sociodemographic, lifestyle, and psychosocial factors up to the age corresponding to the index date. The psychosocial factors (n = 17) experienced in adulthood related to work (interruption, changes, problems), difficulties (academic, financial), judicial problems, marriage or union, divorce or separation (parents’/own), familial conflict, birth of a child, death (spouse or child/other loved one), depression, anxiety, accident, violence or abuse. Potential confounders were based on a priori knowledge. Conditional logistic regression was applied to estimate adjusted odds ratios (ORs) and 95% confidence intervals (CIs) for each psychosocial factor, for CD and UC separately. Multiple imputations were used to address missing data.

**Results:**

Adjusting for potential confounders, the odds of CD were higher among those who had experienced changes in the workplace (OR: 1.58; 95% CI: 0.96-2.61), marriage (OR: 1.32; 95% CI: 1.06–1.64), divorce or separation (OR: 1.75; 95% CI: 1.19-2.58), death of spouse or child (OR: 3.21; 95% CI: 1.12-9.19), anxiety (OR: 2.17; 95% CI: 1.25-3.78), and accident (OR: 1.72; 95% CI: 1.09-2.73). The odds of UC were higher among those who had experienced problems at work (OR: 2.00; 95% CI: 1.03-3.87) and anxiety (OR: 1.75; 95% CI: 0.93 - 3.32) and lower among those exposed to changes in the workplace (OR: 0.48; 95% CI: 0.23-1.00). The other psychosocial factors were not associated with either CD or UC.

**Conclusions:**

Several psychosocial factors, first experienced in adulthood, were associated with increased odds of CD or UC later in life, whereas only one, changes in the workplace was associated with decreased odds of UC. Further research should consider the self-reported subjective impact of these psychosocial factors.

**Funding Agencies:**

CIHRCanada Foundation for Innovation & the Québec Ministry of Education, Leisure and Sports (#12532), Fonds de recherche du Québec-Santé (FRQS, #16227), Multiple Sclerosis Society of Canada (#2435), Institut de la statistique du Québec

